# Validity and feasibility of the self-report EQ-5D-Y as a generic Health-Related Quality of Life outcome measure in children and adolescents with Juvenile Idiopathic Arthritis in Western Cape, South Africa

**DOI:** 10.4102/sajp.v75i1.1335

**Published:** 2019-07-30

**Authors:** Desiree Scott, Christiaan Scott, Jennifer Jelsma, Deepthi Abraham, Janine Verstraete

**Affiliations:** 1 Department of Health and Rehabilitation Sciences, Division of Physiotherapy, Groote Schuur Hospital, Faculty of Health Sciences, University of Cape Town, Cape Town, South Africa; 2 Department of Paediatrics, Paediatric Rheumatology, Red Cross War Memorial Children’s Hospital, University of Cape Town, Cape Town, South Africa; 3 Department of Paediatrics and Child Health, Faculty of Health Sciences, Tygerberg Hospital, University of Stellenbosch, Cape Town, South Africa

**Keywords:** Health-Related Quality of Life, Juvenile Idiopathic Arthritis, EQ-5D-Y, PedsQL, JAMAR, disease severity

## Abstract

**Background:**

Health-Related Quality of Life (HRQoL) data together with clinical findings allow for monitoring of intervention efficacy and the effect on HRQoL. Children with Juvenile Idiopathic Arthritis (JIA) experience symptoms often persisting into adulthood, emphasising the need to track HRQoL.

**Objectives:**

The aim of this study was to investigate psychometric properties of the EuroQol five-dimensional youth questionnaire (EQ-5D-Y) in children with JIA.

**Methods:**

A cross-sectional, analytical study design was used. Children 8 to 15 years were recruited, completing the self-report EQ-5D-Y and two other HRQoL questionnaires. Known group validity was established by comparing the effect size between children with different disease severities. Concurrent validity was tested using Kruskal–Wallis to compare the ranking of scores on different questionnaires. Feasibility was assessed by number of missing responses and time to complete each questionnaire.

**Results:**

All questionnaires were able to distinguish between children with different JIA severity. There was a significant difference in ranking of most Juvenile Arthritis Multidimensional Assessment Report dimension scores across EQ-5D-Y levels, (*p* < 0.05), indicating concurrent validity. There was poor concurrent validity with the PedsQL dimensions tested with EQ-5D-Y, except for ‘pain’ (*p* = 0.001). The EQ-5D-Y was the quickest to complete with no missing values.

**Conclusion:**

This study showed that the EQ-5D-Y is valid and feasible in measuring HRQoL in JIA children and adequately responsive to detect change over time.

**Clinical implications:**

It is quick and easy to use in a busy clinical setting, allowing for effective JIA management monitoring.

## Background

Juvenile Idiopathic Arthritis (JIA) is an autoimmune, chronic rheumatic disorder with episodic flare-ups and remissions, occurring in children 16 years and younger, causing short- or long-term disability (Angeles-Han & Prahalad 2010). The pathophysiology of JIA includes the presence of autoantibodies or autoreactive T cells that initiate and perpetuate an inflammatory response, causing synovial inflammation and hypertrophy and joint effusion. This leads to chronic synovitis with eventual tissue damage and loss of joint function (Prakken, Albani & Martini 2011). Impairments resulting from the joint inflammatory processes and leading to disability include on-going pain, limited range of motion or stiffness, swelling and decreased physical fitness. Other manifestations may include behavioural problems, social isolation, depression and anger (Moorthy et al. 2010). Skeletal growth may be delayed or distorted in severe cases of JIA, as growth hormone levels are low and premature fusion of the epiphyses at the hip may occur (Kwon, Kim & Lee 2015:837; Liem & Rosenberg 2003:664). Rapid advances in the management of JIA, aimed at the elimination of inflammation, reduction of pain and preservation of function, have occurred in the last decade. The availability of disease-modifying antirheumatic drugs (DMARDs) has revolutionised the care of children with JIA in areas where these therapies are accessible. Juvenile Idiopathic Arthritis is, however, still not a curable disease, and access to therapies is limited by cost and shortage of adequately trained healthcare workers (Scott et al. 2018).

Delayed or inaccessible, partially effective or ineffective therapy may result in impairments of structures, physical and psychological functional limitations, all limiting daily activities, which, in turn, may affect the Health-Related Quality of Life (HRQoL) of the child (Adunuri & Feldman 2015). The concept of HRQoL may be defined as the patient’s self-reported perception of the multidimensional impact on their life, of the disease and its management. The dimensions reported on, are generally grouped under physical daily activities, psychological well-being and social interaction (Adunuri & Feldman 2015; Varni et al. 2002).

Disease-specific HRQoL outcome measures, such as the 22-item Paediatric Quality of Life Inventory (PedsQL) Rheumatology Module 3.0 (Varni et al. 2002), assess specific disease-related symptoms in children with rheumatological conditions. The Juvenile Arthritis Multidimensional Assessment Report (JAMAR) (Filocamo et al. 2011) is a composite measure made up of a self-report section on 10 items of HRQoL, and it also includes measurements of 15 functional abilities, joint involvement, disease activity, rating of disease status, and a description of side effects of medications and therapeutic compliance (Filocamo et al. 2011).

Generic HRQoL measures assess general health status across many health conditions. Examples of paediatric generic HRQoL measures are the EuroQol five-dimensional youth questionnaire (EQ-5D-Y), with an overall rating of health on a Visual Analogue Scale (VAS) (Ravens-Sieberer et al. 2010) and the 27-item KIDSCREEN (Ravens-Sieberer et al. 2007). These instruments have been tested in children with JIA and generally found to be useful. The psychometric properties of the disease-specific PedsQL Rheumatology Module have been found to be comparable with the PedsQL Generic Module (Varni et al. 2002). The performance of the JAMAR compared favourably to the PedsQL Rheumatology Module (Filocamo et al. 2011). The generic EQ-5D-Y was found to correlate with the generic Short-Form-36 (SF36) in children with JIA (Wipff et al. 2016). The EQ-5D-Y has been used to assess HRQoL in children with a variety of health conditions, including JIA (April et al. 2012; Burström et al. 2014; Jelsma & Ramma 2010; Scott, Ferguson & Jelsma 2017), but its performance has not been compared to a disease-specific measure in the JIA population.

There are at least three outpatient clinics in Cape Town dedicated to the management of children with JIA.

Children typically attend these outpatient facilities with their parents monthly if they have been started on DMARDs and every 4 months thereafter and biannually or annually if they are stable off DMARDs. The JAMAR is the only outcome measure that is currently used to monitor functional and HRQoL change in the children. However, it is a lengthy questionnaire and not routinely applied. As the joint damage and symptoms affecting HRQoL can persist into adulthood in 50% of children (April et al. 2012), it is important to track HRQoL over the life span. Many of these children tend to default clinics during a period of remission and only return if they relapse, which supports the need to monitor any change in HRQoL. The generic EQ-5D-Y might be appropriate for this use as it can be used until the age of 18 years and, while not interchangeable with the adult EQ-5D, it articulates with this version that could be used in adulthood.

In addition, latent values have been developed for the EQ-5D-Y, which provides a summary score based on the reporting on the five domains (Rivero-Arias et al. 2017). This summary score will later allow for the development of utility weighted values, which will be used for economic analysis, as has already been done for the adult EQ-5D (Grandy & Fox 2008). This is becoming necessary as the cost of treatment is currently high. It was hypothesised that the shorter and quicker EQ-5D-Y, whereas not yielding the details of the child’s condition to the extent of the JAMAR, could be used to track changes in the child’s HRQoL overtime and as a screening for increased inflammatory activity. The JAMAR might then be applied in the children with disease flare or a decrease in HRQoL.

## Aims and objectives

The aim of our study was to investigate the performance (concurrent and known group validity) and feasibility of the EQ-5D-Y as a generic health outcome measure in children and adolescents with JIA.

Specific objectives were to investigate the:

- Concurrent validity by comparing the EQ-5D-Y dimensions with similar dimensions in the JAMAR HRQoL section and PedsQL Rheumatology Module and establishing if there were significant correlations between EQ-5D-Y VAS, EQ-5D-Y latent value, JAMAR HRQoL total score and PedsQL Rheumatology Module total score.
- Known group validity by determining whether the EQ-5D-Y can discriminate between children with different severities of JIA.
- Feasibility by comparing the percentage of missing responses in outcome measures and the time taken to complete each measure.

## Methods

### Study design and setting

An observational, analytical cohort study was used to investigate the performance of the EQ-5D-Y in children and adolescents with JIA. The study was conducted at three outpatient clinics in Cape Town, managing children with JIA. Two clinics were at general tertiary hospitals and the third clinic was at a large paediatric hospital.

### Participants

As two of the outcome measures were only available in English, English-speaking children and adolescents between the ages of 8 and 15 years, diagnosed with JIA by a rheumatologist, were recruited. Children who had successfully passed at least 2 years of formal schooling were recruited to ensure understanding of the VAS and the questions asked in outcome measures. Children attending the clinics with a parent or legal guardian present to sign informed consent were included. Children with an unrelated chronic condition as diagnosed by a medical doctor, poor cognitive function or inability to communicate were excluded.

In total, there were 219 children from 8 to 15 years, registered at the clinics in 2017; however, not all attend the outpatient clinics regularly. The sample size was calculated based on the number of participants required to establish discriminant validity between mild and moderate severity of JIA. It was anticipated that the central limit theorem would apply, and that the one-way ANOVA would be used to compare health profiles between groups. The calculation was based on a root-mean-square standardised effect (RMSSE) of 0.5893, based on an anticipated difference in VAS of 15 between the groups, with a standard deviation of 18 for two groups and a type 1 error rate of 0.05. These values were taken from another study using the EQ-5D-Y in South African children attending a mainstream school and a special school (Jelsma & Ramma 2010). A minimum of 39 children per group were required to ensure a power of 95% for a one-way ANOVA.

### Outcome measures

A self-designed demographic questionnaire and the following HRQoL self-reports were utilised.

#### EuroQol five-dimensional youth questionnaire

The primary outcome measure was the generic EQ-5D-Y. The EQ-5D-Y self-report questionnaire has one item in each of five dimensions: mobility; looking after myself; usual activities; having pain or discomfort; and feeling worried, sad or unhappy. There are three levels of response: 1 = no problems, 2 = some problems or 3 = a lot of problems. In addition, there is a VAS on which subjective, overall health is self-rated, between 0 = worst health imaginable and 100 = best health imaginable.

The EQ-5D-Y has been determined as reliable, valid and feasible in the South African general population and in children with a health condition, between 8 and 16 years (Consolaro et al. 2016; Ravens-Sieberer et al. 2010). The latent scales, developed by Rivero-Arias et al. (2017) for EQ-5D-Y dimensions, were used to determine a summary numeric latent value for the dimension scores.

#### Juvenile Arthritis Multidimensional Assessment Report

The JAMAR, a disease-specific measure, includes self-report on:

- 15 functional ability items, each scored as: 0 = no difficulty, 1 = some difficulty, 2 = much difficulty and 3 = unable to do. Total scores range from 0 to 30.
- Pain, rated on a 21-point VAS, with 0 = no pain, 10 = extreme pain.
- The number of active joints or joint groups, ranging from 0 to 18.
- The level of disease activity, scored on a 21-point VAS, with 0 = no activity, 10 = maximum activity.
- Medication and problems caused by medication.
- School-related problems.
- 10 HRQoL items reported on five physical health and five psychosocial health items. Each item is scored as: never difficult = 0, sometimes difficult = 1, difficult most of the time = 2 and difficult all the time = 3. A total of 0 indicated no difficulty in HRQoL and a total of 30 indicated difficulty every day. The lower the JAMAR HRQoL total score, the better the overall HRQoL.
- The overall rating of well-being (HRQoL) is measured on a 21-numbered VAS, with 0 = very well, 10 = very poorly (Filocamo et al. 2011).

The JAMAR has been found to be feasible and valid for self-report in children with JIA, from 7 to 18 years (Filocamo et al. 2011). Three groups of severity of disease were stratified, based on a composite score of six components of the JAMAR questionnaire. The six components used were functional ability total score, pain score, number of active joints, disease activity level, HRQoL total score and overall rating of well-being. A similar method was used in studies by Filocamo et al. (2011), Ramelet et al. (2017) and the Juvenile Disease Activity Score (JADAS) reviewed by Consolaro et al (2016). As the maximum score for severe disease activity was 108, the three groups were equally divided into this number; therefore, a composite score of 0–36 was used to indicate mild severity of disease, 37–72 moderate severity and 73–108 severe disease activity.

#### Paediatric Quality of Life Inventory Rheumatology module

The disease-specific PedsQL Rheumatology module consists of 22 items in five dimensions: pain and hurt; daily activities; treatment; worry and communication. Scoring is on a five-point Likert scale, 0 = never a problem, 1 = almost never a problem, 2 = sometimes a problem, 3 = often a problem, 4 = almost always a problem and items are summed to give dimension scores. The English PedsQL 3.0 Rheumatology Module was found to be reliable, valid and feasible by Varni et al. (2002). The lower the PedsQL total score, the better the overall HRQoL.

### Procedure

Participants fulfilling the inclusion criteria were recruited during routine check-ups at the rheumatology outpatient clinics.

Data collection took place from May 2017 to April 2018. The purpose and procedure of the study were explained to the children and their parents by one of the authors in face-to-face interviews, while they were waiting at the clinic for their doctor’s appointment. If they indicated a willingness to participate, the parents completed an informed consent form and the children an informed assent form.

The child was then taken to a quiet, secluded area in the clinic and asked to self-complete the EQ-5D-Y, PedsQL and JAMAR outcome measures in an arbitrary order, with the author available to assist with clarification of the questionnaires if necessary. Neither the author nor the parent prompted or guided the child’s reporting. The parent was asked to assist with the prescribed medication, as recorded on the JAMAR, if the child was not able to recall the medication used. The time taken to complete each of the questionnaires was recorded by the author.

If the child was called for their doctor’s appointment before completing all the questionnaires, the interview was put on hold. The author encouraged the child and parent to return after the doctor’s appointment to complete the questionnaires; however, they were not forced to do so. Incomplete forms were shredded.

Because of the small pool of children with JIA attending the weekly clinic visits, the proposal was amended and further ethical approval sought to allow the authors to contact participants on the JIA database telephonically and invite them to attend an information sharing session. During this workshop type session, for which only seven participants arrived, information on the physiotherapy management of JIA, the importance of exercise and diet was shared, with participants asking questions and giving useful advice to each other. A short exercise programme including dancing and yoga followed. The attendees were then invited to participate in the study, following the same procedure as for the children at the JIA clinics. A light snack was offered to all the attendees and they were compensated for their travel costs.

### Ethical considerations

Ethical approval was granted by the Human Research Ethics Committee (HREC) of the Faculty of Health Sciences, University of Cape Town (UCT) (HREC REF: 209/2017) (Appendix 1) and reciprocal approval from Stellenbosch University Human Research and Ethics Committee (N18/01/008_RECIP_UCT 209/2017). Permission from the Department of Health to conduct nontherapeutic research with minors was sought and permission to conduct the study at the three research facilities was obtained.

One child was referred to Child Psychiatry because of non-compliance with medication, not keeping up with school work and for being a victim of bullying at school. Another child and parent were referred to Child Psychiatry for counselling because of non-compliance with medication.

### Data analyses and management

Descriptive statistics were used to describe the sample. Although the sample was large enough for the central limit theorem to apply, the data were grossly non-normal and non-parametric statistics were used for ordinal and numerical data. A summary latent value was determined using utility scales for EQ-5D-Y dimensions (Rivero-Arias et al. 2017). The disease severity was determined by a composite score of six components on the JAMAR, with the cut-off points as described earlier. Known group validity was established by comparing mean ranking of total scores and effect size (ES), Cohen’s *d*, between children with mild or moderate severity levels of JIA. Concurrent validity was tested using Kruskal–Wallis to determine whether there was a significant difference between the relevant JAMAR and PedsQL scale items across the different levels of comparable EQ-5D-Y dimensions. Correlation between EQ-5D-Y VAS, latent value, JAMAR and PedsQL total scores were compared using Spearman’s rho. Feasibility was assessed by determining the number of missing responses and time taken to complete each questionnaire.

## Results

There were 219 children with JIA on the hospital databases and thus eligible for recruitment. Of these, 66 children were either recruited at the outpatient clinics or while attending an information sharing session, during the 1-year data collection period. Two children’s information was withdrawn, as their parents declined consent for the child to continue after the clinic visit, leaving 64 participants.

Of the 64 participants, 40 (62.5%) were female. The mean age was 11.8 (± 2.53) years.

On the JAMAR outcome measure cut-off points, 54 participants (84.4%) fell within the mild disease category and 10 (14.1%) within the moderate severity category. Only a single participant was severely affected and was excluded from analysis.

### Profiles of children’s Health-Related Quality of Life using EuroQol five-dimensional youth questionnaire, Juvenile Arthritis Multidimensional Assessment Report Health-Related Quality of Life and Paediatric Quality of Life Inventory

**FIGURE 1:**
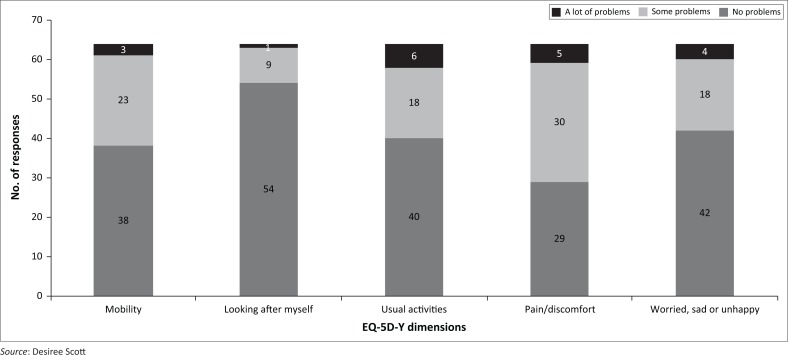
Number of problems reported in each level of EuroQol five-dimensional youth (EQ-5D-Y) questionnaire. *N* = 64.

The highest number of problems was reported in the EQ-5D-Y pain/discomfort dimension (54.7%). Slightly more problems were reported in mobility (40.6%) compared to usual activities (37.5%) and worried, sad or unhappy (34.4%). The least problems were reported in looking after myself (15.6%) (Figure 1).

The mean (SD) for EQ-5D-Y VAS score, of overall health, was 77.8 (± 23.22).

The EQ-5D-Y latent values (summary score of dimensions) were not normally distributed and non-parametric tests were used. The median for latent values was -1.515 (range: -7.9 to 0). The lower the score, the worse the health state.

**FIGURE 2:**
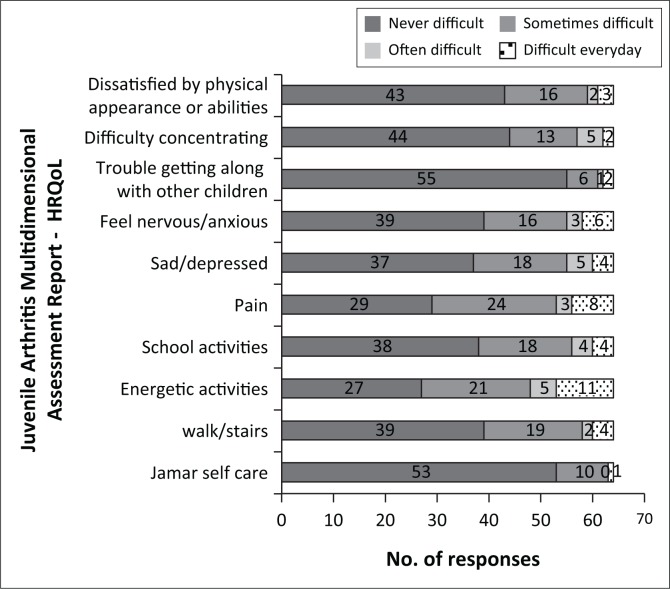
Number of responses in each level of Juvenile Arthritis Multidimensional Assessment Report Health-Related Quality of Life. *N* = 64.

Using the JAMAR HRQoL, the highest number of problems was reported in the ‘Doing energetic activities, such as running, playing football, etc.’ dimension (57.8%) and ‘pain’ (54.7%), followed by problems in ‘feeling sad or depressed’ (42.2%) and ‘walking and climbing stairs’, ‘doing school activities’, ‘nervous or anxious’ (39.1%). The dimensions with the least reported problems were ‘getting along with other children’ (14.1%) and ‘self-care’ (17.2%) (Figure 2).

In the PedsQL R heumatology Module, the highest number of problems was reported in the ‘Worry about illness’ dimension (68.8%), ‘Ache in joints and muscles’ and ‘Worry about medicines working’ (both 65.6%), followed by ‘Hard to explain my illness to other’ (64.1%) and ‘Morning stiffness’ (61%).

**FIGURE 3:**
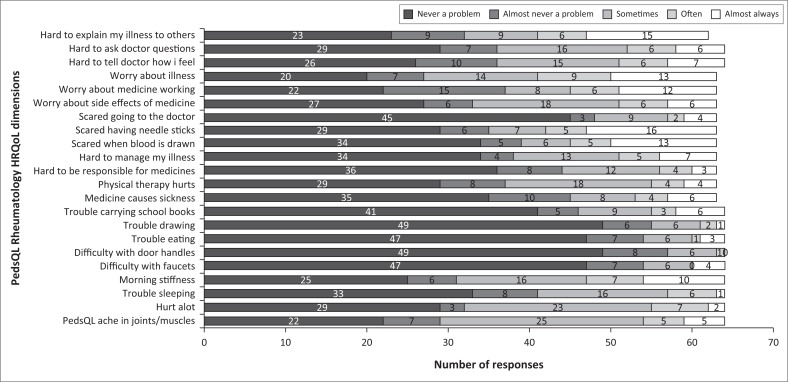
Number of responses in each level of Paediatric Quality of Life Inventory Rheumatology Module. *N* = 64.

The least problems were reported in upper limb functions such as ‘Difficulty with drawing’, ‘Difficulty with door handles’ and ‘Eating’ (23.3% and 26.6%) (Figure 3).

### Concurrent validity

Kruskal–Wallis was used to test independence in the relevant JAMAR and PedsQL scale items with the different levels of comparable EQ-5D-Y domains. When there were no scores for a particular problem level (1, 2 or 3) on the independent EQ-5D-Y variable, this level was excluded, and the Mann–Whitney *U* test was used to compare the remaining two levels.

Table 1a indicates that there was a significant difference in ranking of all comparable JAMAR dimension scores across the three EQ-5D-Y levels, (*p* < 0.05), except for the ‘anxiety’ dimension (*p* = 0.072).

**TABLE 1a:** Difference in composite scores of the relevant Juvenile Arthritis Multidimensional Assessment Report scale items across the three different levels of comparable EuroQol five-dimensional youth questionnaire dimensions.

EQ-5D-Y dimension	JAMAR item	Kruskal–Wallis H value	*p*	Mann–Whitney *U*	*p*
Mobility	Difficulty walking/stairs	16.37	0.001*	-	-
Looking after myself†	Self-care	-	-	−3.23	0.001*
Usual activities	School activities and playing with friends	15.64	0.011*	-	-
Pain	Pain	32.09	< 0.001	-	-
Worried, sad or unhappy	Nervous/anxious	7.01	0.072	-	-

JAMAR, Juvenile Arthritis Multidimensional Assessment Report; EQ-5D-Y, EuroQol five-dimensional youth questionnaire. H(3, n:64).

† , only two levels reported.

* , Statistically significant (p < 0.05).

There was poor concurrent validity with the PedsQL dimensions tested with the different levels of the EQ-5D-Y, except for ‘pain’, which indicated a significant difference in ranking of PedsQL ‘pain subtotal’ across the levels of EQ-5D-Y ‘pain’ (*p* = 0.001) (Table 1b).

**TABLE 1b:** Difference in composite scores of the relevant Paediatric Quality of Life scale items across the three different levels of comparable EuroQol five-dimensional youth questionnaire dimensions. *n* = 64.

EQ-5D-Y dimension	PedsQL item	Kruskal–Wallis H value	*p*
Usual activities	Daily activities subtotal	H(12) = 17.47	0.133
Pain or discomfort	Pain subtotal	H14) = 36.67	0.001*
Worried, sad or unhappy	Worry subtotal	H(12) = 17.00	0.149

EQ-5D-Y, EuroQol five-dimensional youth questionnaire; PedsQL, Paediatric Quality of Life Inventory.

* , Statistically significant (p < 0.05).

Table 2a demonstrates that the JAMAR total score was significantly correlated with the other three outcome measures and the correlation with the latent value was marginally the highest. The VAS was significantly negatively correlated with age in females (Table 2c), which implies that older females reported a worse HRQoL and the latent value was significantly positively correlated with age in males (Table 2b), indicating that older males reported fewer problems on the dimensions.

**TABLE 2a:** Correlations between EuroQol five-dimensional youth questionnaire Visual Analogue Scale, latent value and Juvenile Arthritis Multidimensional Assessment Report Health-Related Quality of Life total score and Paediatric Quality of Life Inventory Rheumatology Module total score, for males and females, including age. *n* = 64.

Variable A	Variable B	Correlation coefficient (*r*)	*p*
EQ-5D-Y latent value	EQ-5D-Y VAS	0.39	0.001*
EQ-5D-Y latent value	JAMAR HRQoL total score	−0.66	< 0.001*
EQ-5D-Y latent value	PedsQL total score	−0.70	< 0.001*
EQ-5D-Y latent value	Age	0.11	0.369
EQ-5D-Y VAS	JAMAR HRQoL total score	−0.38	0.001*
EQ-5D-Y VAS	PedsQL total score	−0.32	0.010*
EQ-5D-Y VAS	Age	−0.20	0.121
JAMAR HRQoL total score	PedsQL total score	0.64	< 0.001*

* , Spearman’s rho correlations statistically significant (*p* < 0.05).

**TABLE 2b:** Correlations between outcome measures for males. *n* = 24.

Variable A	Variable B	Correlation Coefficient (*r*)	*p*
EQ-5D-Y latent value	EQ-5D-Y VAS	0.55	0.005*
EQ-5D-Y latent value	JAMAR HRQoL total score	−0.53	0.007*
EQ-5D-Y latent value	PedsQL total score	−0.56	0.005*
EQ-5D-Y latent value	Age	0.45	0.025*
EQ-5D-Y VAS	JAMAR HRQoL total score	−0.45	0.027*
EQ-5D-Y VAS	PedsQL total score	−0.36	0.080
EQ-5D-Y VAS	Age	0.05	0.235
JAMAR HRQoL total score	PedsQL total score	0.49	0.016*

* , Spearman’s rho correlations statistically significant (*p* < 0.05).

**TABLE 2c:** Correlations between outcome measures for females. *n* = 40.

Variable A	Variable B	Correlation coefficient (*r*)	*p*
EQ-5D-Y latent value	EQ-5D-Y VAS	0.29	0.069
EQ-5D-Y latent value	JAMAR HRQoL total score	−0.67	< 0.001*
EQ-5D-Y latent value	PedsQL total score	−0.71	< 0.001*
EQ-5D-Y latent value	Age	−0.05	0.770
EQ-5D-Y VAS	JAMAR HRQoL total score	−0.33	0.036*
EQ-5D-Y VAS	PedsQL total score	−0.23	0.154
EQ-5D-Y VAS	Age	−0.40	0.011*
JAMAR HRQoL total score	PedsQL total score	0.65	< 0.001*

JAMAR, Juvenile Arthritis Multidimensional Assessment Report; PedsQL, Paediatric Quality of Life Inventory; VAS, Visual Analogue Scale; HRQoL, Health-Related Quality of Life.

* , Spearman’s rho correlations statistically significant (*p* < 0.05).

### Known group validity and responsiveness

The disease severity of the participants was categorised into mild or moderate using a composite score of six components of the JAMAR as described above.

Effect size was determined using Cohen’s *d*.

Cohen’s *d* = (*M*_2_ – *M*_1_) ⁄ SD_pooled_

All measures were able to distinguish between children with mild and moderate severity of JIA and the ESs were medium to large. The PedsQL demonstrated the highest known group validity (Table 3).

**TABLE 3:** Comparison of scores across the mild and moderate levels of disease severity.

Variable	Mean rank, mild, *n* = 54	Mean rank, moderate, *n* = 10	*p*	ES, Cohen’s *d*	Interpretation
JAMAR HRQoL total	28.92	51.85	< 0.001*	1.72	Large
PedsQL total	28.19	55.75	< 0.001*	1.87	Large
EQ-5D-Y VAS	34.46	21.9	0.047*	0.72	Medium
EQ-5D-Y latent value	35.19	17.95	0.006*	1.46	Large

Interpretation of Cohen’s d: small effect (0.2), medium effect (0.5) and large effect (0.8) (http://rpsychologist.com/d3/cohend/).

HRQoL, Health-Related Quality of Life; JAMAR, Juvenile Arthritis Multidimensional Assessment Report; EQ-5D-Y, EuroQol five-dimensional youth questionnaire; PedsQL, Paediatric Quality of Life Inventory; VAS, Visual Analogue Scale.

* , Statistically significant (p < 0.05).

### Feasibility

The EQ-5D-Y took just over 1 min to complete, approximately half as much time as the PedsQL. The JAMAR took the longest to complete (Table 4).

**TABLE 4:** Time taken to complete the measures (in minutes). *n* = 64.

Variable	Mean	SD	*n*	Time not recorded in (*n*) questionnaires
EQ-5D-Y	1.3	0.81	61	3
JAMAR	8.9	3.54	61	3
PedsQL	2.7	1.32	62	2

JAMAR, Juvenile Arthritis Multidimensional Assessment Report; EQ-5D-Y, EuroQol five-dimensional youth questionnaire; PedsQL, Paediatric Quality of Life Inventory.

There were no missing responses in any dimensions of the outcome measures. Time to complete was not recorded in eight questionnaires.

## Discussion

The primary aim of the study, to determine the validity of the EQ-5D-Y in children with JIA, was accomplished and there is evidence that the shorter, generic instrument measures the same constructs as the longer, disease-specific measures (Tables 1 and 2). In addition, the dimensions, as summarised in the EQ-5D-Y latent value, could detect a large ES between known group effect and the EQ-5D-Y VAS, a medium ES (Table 3).

During the 1-year period, only 66 children registered at the clinics attended. With the advances in the medical drug management of JIA over the last 20 years, children now experience fewer symptoms of the disease. There has been a decrease in the level of disease activity and pain experienced, as well as an improvement in health professional’s global assessment of the disease, because of the improved drug management (Carle, Dewitt & Seid 2011; Michel et al. 2009; Pruunsild et al. 2007). As a result, most participants (84.4%) fell within the mild disease category. A German study conducted between 2010 and 2012 found that with improved rheumatologic care, newly diagnosed JIA patients reported a similar psychosocial health status to their healthy peers and only slightly worse physical health (Listing et al. 2018).

Most of the participants were female (62.5%), similar to other studies in Western populations (Filocamo et al. 2011; Freire & Ferreira 2018; Lovell et al. 2008). As reported in other studies (Haverman et al. 2012; Heiligenhaus et al. 2013; Sawyer et al. 2003), older females reported significantly worse overall HRQoL on the VAS, while the older males reported better overall HRQoL. Similarly, when comparing EQ-5D-Y latent values females reported significantly worse on dimensions compared to males. A similar trend was observed when reporting on the JAMAR and the PedsQL (Table 2). Other studies have reported that moods and emotions, self-perception and social acceptance seem to influence females’ reported HRQoL (Heiligenhaus et al. 2013; Jelsma & Ramma 2010; Sawyer et al. 2003), which could contribute towards females with JIA reporting lowered HRQoL and not necessarily only because of the impact of the disease. As the EQ-5D-Y was able to demonstrate these trends, it would be useful to do a prospective study to determine why the self-reported health state seems to deteriorate in females but improves in males.

As expected, participants reported most problems on the EQ-5D-Y pain/discomfort dimension (57.4%), followed by problems in mobility (40.6%) and usual activities (37.5%) (Figure 1). Pain and functional ability have been found to be the main predictors of HRQoL in children with JIA (April et al. 2012; Haverman et al. 2012; Sawyer et al. 2003). The EQ-5D-Y latent value correlated significantly with the VAS, but the correlation was somewhat low (Table 2). In the light of previous studies indicating that children with chronic disease report relatively high VAS, despite recognising their functional problems, this result is not too surprising (Jelsma & Ramma 2010; Otto et al. 2018; Scott et al. 2017).

Children reported most problems on similar dimensions on the JAMAR, but slightly more problems with ‘doing energetic activities’ dimension (57.8%) than ‘pain’ (54.7%) (Figure 2). As children would be expected to have greater problems with the more intense JAMAR ‘running and playing football’ than with EQ-5D-Y ‘walking’, it is not surprising that they reported most problems on this dimension in the JAMAR.

Reporting on the PedsQL Rheumatology Module, however, was highest in ‘worry’ (68.8%), followed by pain ‘ache in joints and muscles’ (65.6%) (Figure 3). The focus of reporting on the PedsQL Rheumatology Module is somewhat different to the other two instruments as there are no questions specifically relating to mobility and the ‘daily activities’ section focuses almost entirely on upper limb function. In addition, the ‘worry’ items relate specifically to the disease and its management. This could imply that the JAMAR and the PedsQL measure slightly different constructs and explain the minimally lower rho between the two when compared to the JAMAR and the EQ-5D-Y latent value.

The scores in related dimensions of the other instruments differed significantly across the three EQ-5D-Y levels, which would indicate that similar constructs were measured. This was true of all dimensions tested, except, surprisingly, the ‘worried, sad or unhappy’ and the JAMAR ‘nervous/anxious’ item (Table 1). It could be that the children do not understand the concept of anxiety as well as they do sadness or unhappiness, and this should be investigated further.

All measures were able to distinguish between mild and moderate severity of JIA, but the PedsQL did yield the greatest ES (Table 3). This may be as a result of the larger number of items included in the instrument and it remains to be seen whether the newly developed EQ-5D-Y 5L, as opposed to the Y-3L used here, will result in an instrument more responsive to differences.

The EQ-5D-Y took the shortest time to complete, but the PedsQL also took less than 3 min (Table 4). Currently, the JAMAR is occasionally used in the JIA outpatient clinics, but as it is time-consuming to complete, it is often not done. There were no missing values in any of the outcome measures, as the authors reminded the participants to complete all questions.

### Study limitations

The major limitation of our study is the small sample size. This may well have reduced the power to detect relationships. Also, difference in methodology and engagement with the seven participants who attended the information sharing session may have improved their compliance and altered short-term HRQoL. The positive experience of this small group of children during the session may have inflated HRQoL reporting, but as most tests yielded the expected significant results, this may not have had an impact on the conclusions that can be drawn from the results.

## Conclusion and recommendations

The generic EQ-5D-Y performed well when compared to the disease-specific outcome measures in the disease area of JIA, and it is suggested that it could be used with confidence in the context of JIA outpatient clinics. The EQ-5D-Y can contribute valuable information particularly on pain and mobility and overall HRQoL in JIA children, assisting health practitioners to develop appropriate and holistic interventions.

Should the child’s health state deteriorate, as determined by the EQ-5D-Y, it is recommended that the lengthier, disease-specific JAMAR be used to fully explore the impact of the disease with regard to the side effects of medication, site and nature of pain and functional limitations.

The PedsQL Rheumatic Module showed a large ES in discriminating between the known groups and is clearly a useful instrument for monitoring change in children with JIA. It was also quick to administer.

However, the disadvantage of the PedsQL is that the functional component focusses on upper limb activities only. In addition, it is not generic and thus cannot be used to compare children with other health conditions. There is also no composite score based on societal preferences that can be used for economic evaluation, whereas the development of a utility index is underway for the EQ-5D-Y. The EQ-5D-Y has been translated and is available in over 120 languages, including Afrikaans and isiXhosa versions, allowing for inter-cultural and international comparisons of JIA data.

Based on these results, the use of the EQ-5D-Y is recommended as a routine outcome measure of HRQoL, providing a baseline measure and to be administered during each clinic visit, in the appropriate language of the child. Continued tracking of the HRQoL of the children over time would aid in monitoring the overall performance of the clinic and any future changes in management strategy. The imminent development of the utility index will make cost-effective analysis possible in the near future.
